# HqiA, a novel quorum-quenching enzyme which expands the AHL lactonase family

**DOI:** 10.1038/s41598-017-01176-7

**Published:** 2017-04-19

**Authors:** Marta Torres, Stéphane Uroz, Rafael Salto, Laure Fauchery, Emilia Quesada, Inmaculada Llamas

**Affiliations:** 1 Department of Microbiology, Faculty of Pharmacy, University of Granada, Granada, Spain; 2 Institute of Biotechnology, Biomedical Research Center (CIBM), University of Granada, Granada, Spain; 3 UMR 1136 INRA-Université de Lorraine Interactions Arbres-Microorganismes, Centre INRA de Nancy, Champenoux, France; 4 Department of Biochemistry and Molecular Biology II, Faculty of Pharmacy, University of Granada, Granada, Spain

## Abstract

The screening of a metagenomic library of 250,000 clones generated from a hypersaline soil (Spain) allowed us to identify a single positive clone which confers the ability to degrade *N*-acyl homoserine lactones (AHLs). The sequencing of the fosmid revealed a 42,318 bp environmental insert characterized by 46 ORFs. The subcloning of these ORFs demonstrated that a single gene (*hqiA*) allowed AHL degradation. Enzymatic analysis using purified HqiA and HPLC/MS revealed that this protein has lactonase activity on a broad range of AHLs. The introduction of *hqiA* in the plant pathogen *Pectobacterium carotovorum* efficiently interfered with both the synthesis of AHLs and quorum-sensing regulated functions, such as swarming motility and the production of maceration enzymes. Bioinformatic analyses highlighted that HqiA showed no sequence homology with the known prototypic AHL lactonases or acylases, thus expanding the AHL-degrading enzymes with a new family related to the cysteine hydrolase (CHase) group. The complete sequence analysis of the fosmid showed that 31 ORFs out of the 46 identified were related to *Deltaproteobacteria*, whilst many intercalated ORFs presented high homology with other taxa. In this sense, *hqiA* appeared to be assigned to the *Hyphomonas* genus (*Alphaproteobacteria*), suggesting that horizontal gene transfer had occurred.

## Introduction

Many microorganisms use a genetic regulatory mechanism to monitor their cell density and to establish a coordinated behaviour^1–3^. This regulation mechanism, initially identified in bacteria, has been termed quorum sensing (QS). It is based on the production of diffusible signal molecules (also called autoinducers) by bacteria, which accumulate in their surrounding environment and once they reach a threshold concentration allow the induction or repression of numerous genes^2^. To date, different types of signal molecules have been identified, but the most correspond to the class of the *N*-acylhomoserine lactones (AHLs)^4, 5^. AHL-based communication systems have been studied extensively and identified as playing a crucial role in the regulation of several functions in terrestrial and aquatic environments. Indeed, the production of exoenzymes in *Pectobacterium carotovorum*, motility of *Aeromonas hydrophila*, luminescence of *Aliivibrio fischeri*, antibiotic biosynthesis by *Pseudomonas aeruginosa* and conjugative transfer of plasmid Ti in *Agrobacterium tumefaciens* all involve an AHL-based regulation mechanism^3, 6, 7^. Nevertheless, little information is known about the QS systems of halophilic bacteria and even less on hitherto uncultivated microorganisms. A few studies based on a non-cultivation metagenomic approach have been carried out and new LuxR/LuxI-type QS systems have been identified from uncultivated microorganisms in activated sludge and forest soils^8, 9^. Regarding extreme environments, such as hypersaline soils, QS systems have only been described in cultivable bacteria belonging to the *Halomonadaceae* family^10, 11^. In this context, our understanding of QS signal communication between bacteria living in such extreme environments remains limited.

Besides the ability to produce and use AHL-based communication systems, the ability to inhibit QS-regulated functions or to degrade AHLs was also reported for very different organisms^5^. Such abilities have been described for bacteria, fungi, plants and animals, suggesting that all these organisms have developed various mechanisms to interfere with or disrupt these cellular communication systems^5, 12–16^. One of the first mechanisms historically described is related to the production of chemical compounds (quorum sensing inhibitors or QSI), acting as antagonists and interfering with the transcriptional regulator structure^17–19^. Another mechanism that strongly perturbs or even abolishes QS-regulated functions is related to the production of enzymes capable of degrading the AHL-signal molecules, known as quorum quenching (QQ)^5^. To date, 3 different groups of enzymes have been identified according to the enzymatic mechanism involved: the AHL lactonases (lactone hydrolysis), the AHL acylases (amide hydrolysis) and the AHL oxidases/oxidoreductases (oxidoreduction)^16, 20–22^. Indeed, very different AHL lactonase types and AHL acylase types have been identified, suggesting that convergent evolution events occurred and that other unrelated types may exist in the environment^5^. To date, these different types of AHL-degrading enzymes have been identified in very different lineages of bacteria, such as *Acidobacteria*, *Actinobacteria*, *Bacteroidetes*, *Firmicutes* and *Proteobacteria*, by using cultivation-dependent approaches and for some of them the related genes have been characterized^22–27^. Only some of the known genes encoding AHL-degrading enzymes have been characterized using a cultivation-independent approach based on the construction and screening of metagenomic libraries constructed from soil and marine environments^28, 29^. The construction of such libraries allows the screening of DNA from cultivated and hitherto uncultivated organisms from different environments, thus increasing the possibility to identify new genes of interest. Such an approach gives the opportunity to develop interfering processes based on the inhibition of AHL perception or AHL degradation, and it opens up new perspectives in industrial, medical and agronomic fields as QQ represents an environmentally friendly alternative for the use of chemicals and antibiotics. Notably, QQ has, among its main biotechnological applications, the ability to control biofouling and the regulation of bacterial pathogens.

In this study, we have constructed and screened a metagenomic library from a hypersaline soil to identify potential genes involved in QS or in QQ. To the best of our knowledge, AHL-producing halophilic bacteria have so far only been identified in hypersaline soils by cultivation-dependent methods and no AHL-degrading bacteria have been reported in these extremophilic environments^10, 11^. By using a fast screening method based on pooled clones rather than on the screening of individual clones, we were able to screen a fosmid library of 250,000 clones of *E*. *coli* for the ability to produce or degrade AHLs. This screening allowed the identification of one clone capable of degrading AHLs. Among the 42 kb of the fosmid insert, one ORF (*hqiA*) was identified and biochemically characterized by HPLC/MS as coding for an AHL lactonase. The analysis of the protein sequence revealed no homology with the well-known AHL-degrading enzymes, showing that HqiA represents a new type of enzyme in the AHL lactonase family. A detailed analysis revealed that it clustered into the cysteine hydrolase (CSHase) group and showed strong homology with isochorismatase-like and *N*-carbamoylsarcosine amidase-like enzymes. Moreover, characterization of the neighbouring ORFs allowed us to hypothesize the phylogenetic belonging of the fosmid DNA insert and to suggest that *hqiA* was inherited from a horizontal transfer.

## Results

### Screening of AHL-degrading genes from a metagenomic library

**Figure 1:**
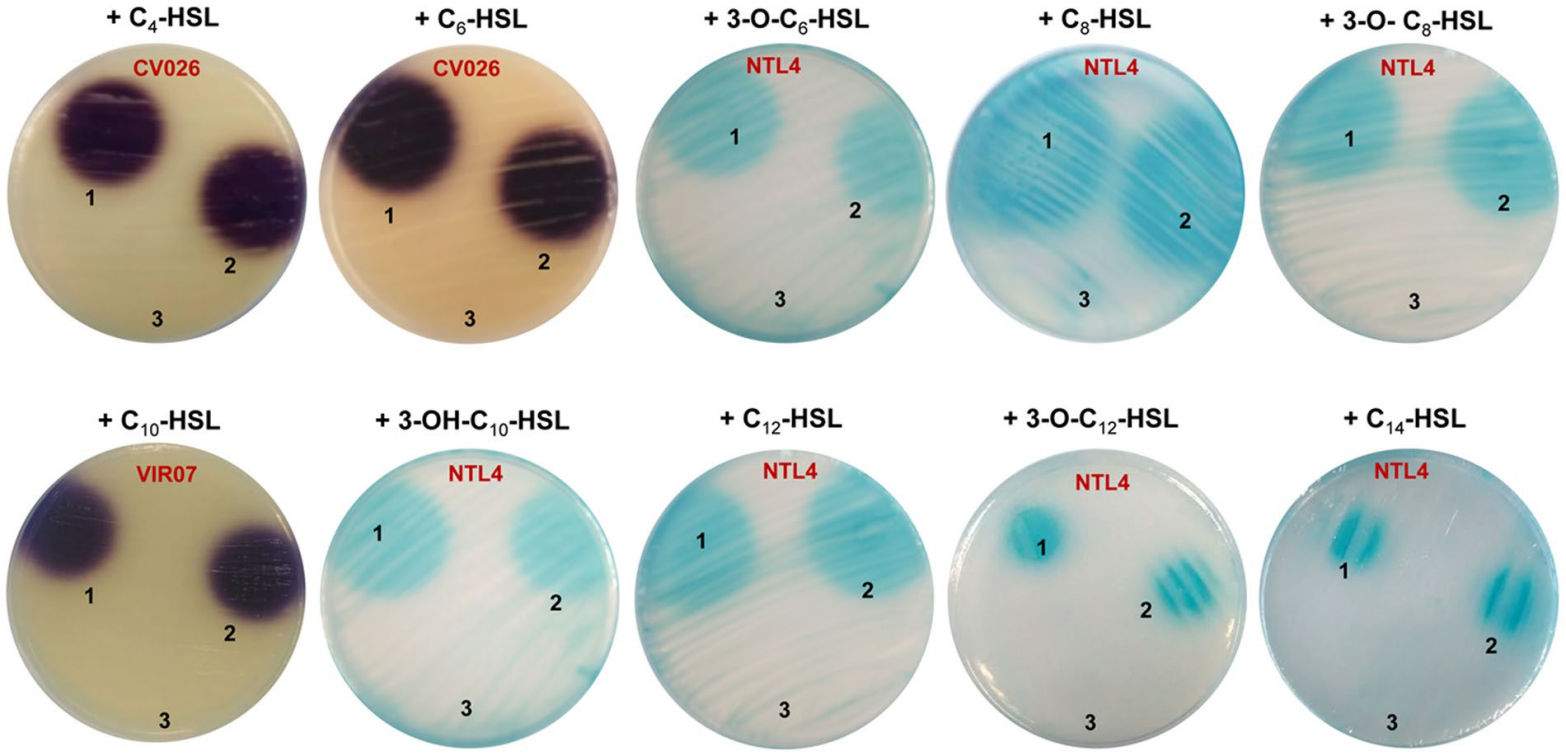
Diffusion agar-plate assay to detect AHL degradation using the biosensors *Chromobacterium violaceum* CV026, *C*. *violaceum* VIR07 and *Agrobacterium tumefaciens* NTL4 (pZLR4). Cell-free LB medium (1), *E*. *coli* S17 λ *pir* (2) and *E*. *coli* S17 λ *pir* harbouring the fosmid f10/17.1H (3) were supplemented with the same quantities of C_4_-HSL, C_6_-HSL, 3-O-C_6_-HSL, C_8_-HSL, 3-O-C_8_-HSL, C_10_-HSL, 3-OH-C_10_-HSL, C_12_-HSL, 3-O-C_12_-HSL and C_14_-HSL.

**Figure 2:**
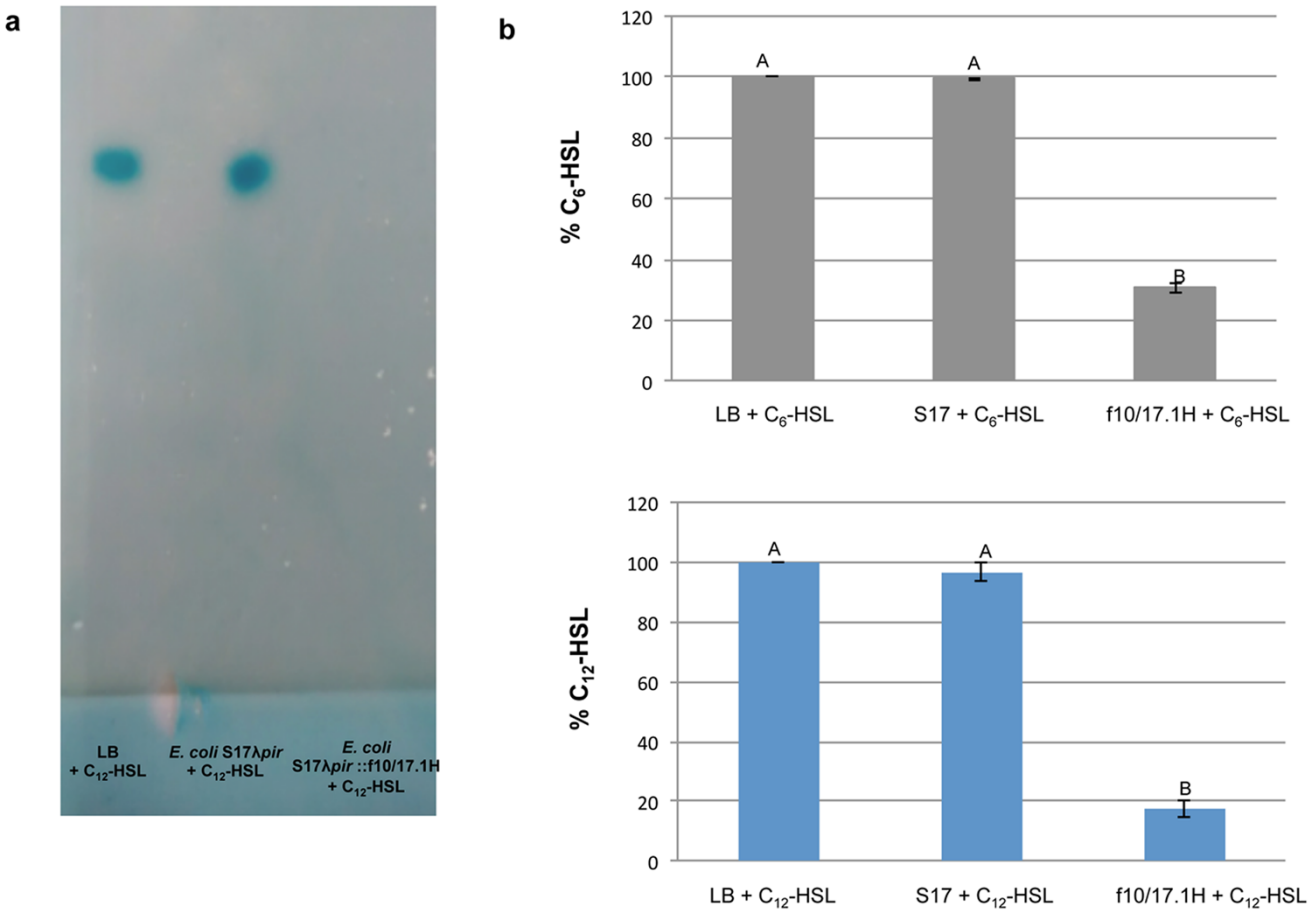
Confirmation of AHL-degradation activity. (**a**) TLC analysis using the biosensor *Agrobacterium tumefaciens* NTL4 (pZLR4). (**b**) HPLC/MS measurements of remaining C_6_-HSL and C_12_-HSL after 24 h incubation with *E*. *coli* S17 λ *pir* and *E*. *coli* S17 λ *pir*::f10/17.1H. LB medium was used as control. Initial AHL concentration was 25 µM. Error bars represent standard deviations. Different letters above the bars indicate that the values are significantly different according to a one-factor (treatment) ANOVA (P < 0.05) and the Tukey test.

Environmental DNA extracted from a hypersaline soil located in the Finca La Salina (Rambla Salada, Murcia, Spain) was used to construct a metagenomic library. This library consists of 250,000 metagenomic fosmid clones each containing a DNA fragment of ~40 kb as determined by enzymatic restriction on randomly sub-sampled clones. The screening of the metagenomic library was done using a pool strategy, which consisted of the conditioning of the metagenomic library to obtain a theoretical number of 50 clones per well (4,800 clones per 96-well microplate). Using this strategy, a screening based on the appropriate AHL biosensor was done to identify AHL-producing or AHL-degrading clones. After a two-day incubation without AHL addition, none of the wells of the 52 microplates screened was positive for AHL production. On the contrary, after a two-day incubation with C_6_-HSL, one well did not activate the *Chromobacterium violaceum* CV026 biosensor. To identify the clone(s) involved, serial dilutions were made from the positive well to obtain individual colonies. The posterior screening of the QQ activity of these colonies allowed the identification of 17 positive clones, thus conferring to *Escherichia coli* the ability to degrade C_6_-HSL. The enzymatic restriction analyses revealed that the different clones recovered corresponded to a unique fosmid, which we named f10/17.1H (data not shown). This fosmid was purified and transferred into *E*. *coli* S17 λ *pir* to carry out additional AHL-degradation assays. These assays revealed that this fosmid conferred the ability to degrade a broad range of unsubstituted, oxo- and hydroxyl-substituted AHLs to *E*. *coli* (Fig. 1). To identify whether the phenotype observed was related to degradation or inhibition, TLC and HPLC/MS analyses were carried out. Based on AHL-degradation assays performed with short- and long-chain AHLs (C_6_-HSL and C_12_-HSL), both TLC and HPLC/MS analyses revealed that the activity corresponded to AHL degradation and not to an inhibition of the biosensor (Fig. 2). Notably, the HPLC/MS analyses confirmed that *E*. *coli* S17 λ *pir* carrying the fosmid f10/17.1H significantly degraded AHLs compared to the control *E*. *coli* S17 λ *pir* (P < 0.0001). Comparison of the C_6_-HSL and C_12_-HSL degradation assays also revealed that after 24 h of incubation, a significantly higher efficacy of degradation was obtained for C_12_-HSL (Fig. 2B, P < 0.0001).

### *In silico* analysis of the f10/17.1H fosmid DNA sequence

**Figure 3:**
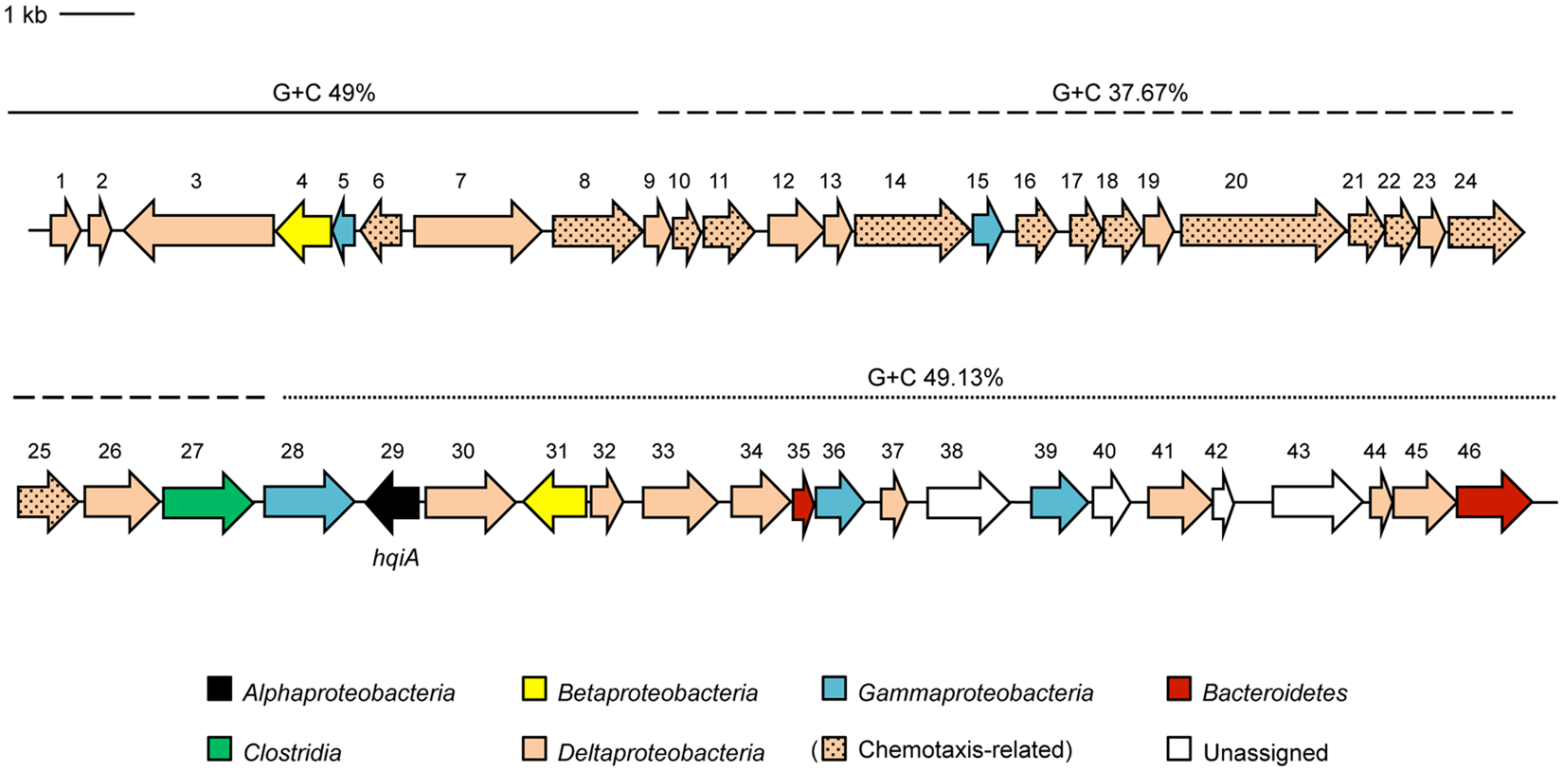
Genetic map of the DNA insert of the fosmid f10/17-1H. The 46 ORFs are numbered and coloured according to their phylogeny, ORF29 being (in black) *hqiA*. The chemotaxis related genes are also presented as square dots.

The *in silico* analysis of the 42,318 bp environmental DNA revealed that it contained 46 open reading frames (ORF) in both orientations (Fig. 3). The analysis of the G + C content with GC-Profile highlighted a global G + C of 45.6 mol % (100 bp window), with three segments (1–8,963 bp: 49 mol %; 8,926–21,987 bp: 37.7% and 21,958–42,318 bp: 49.1 mol %) (Fig. 3). Besides these variations of the G + C content, a detailed analysis of the sequence revealed that the codon usage of most of the amino acids detected was equally distributed among their different forms.

**Figure 4:**
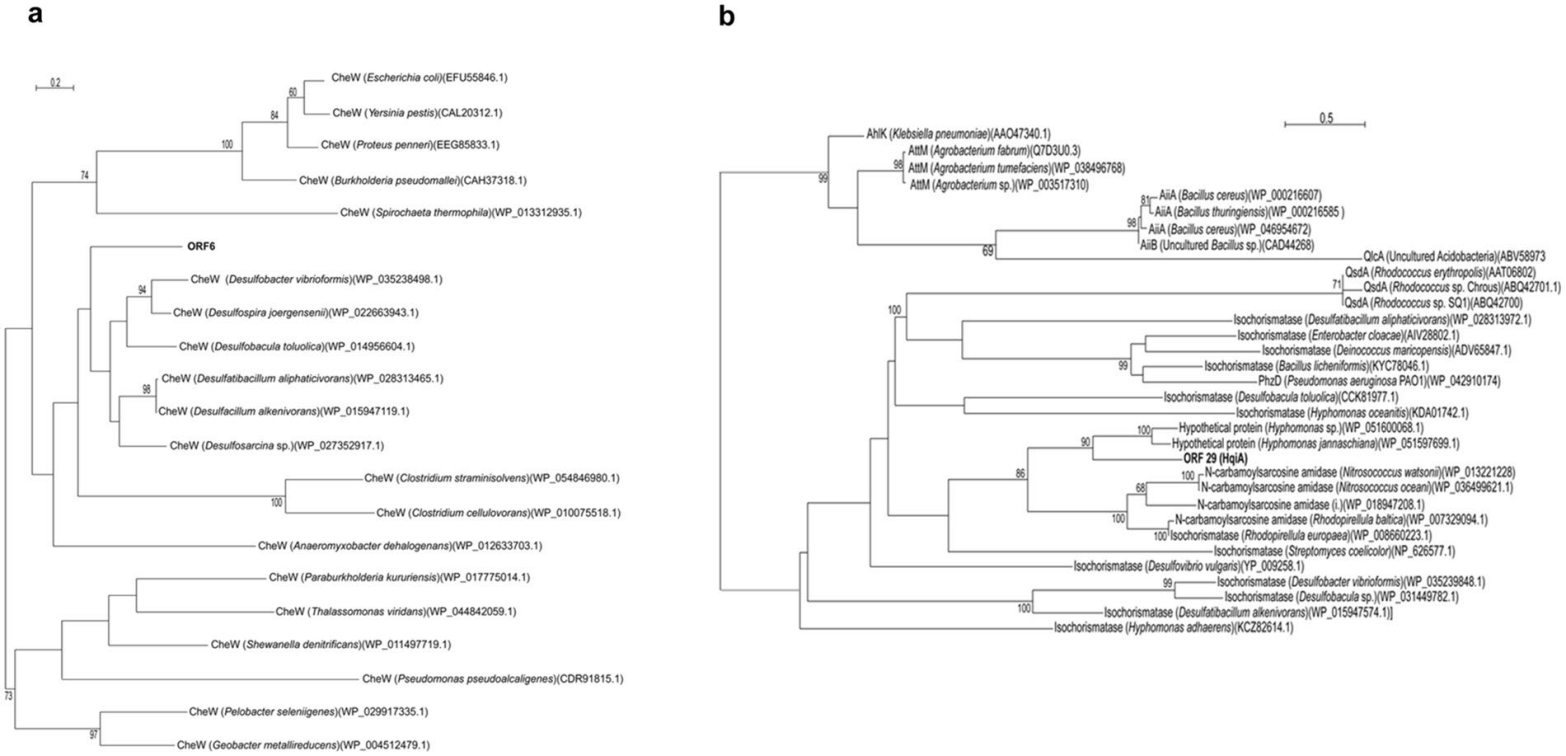
Phylogenetic analysis of the ORFs found in fosmid f10/17.1H based on protein sequences. (**a**) ORF6 as an example of affiliation of ORFs by phylogenetic analysis of representative proteins of each genus identified based on maximum likelihood (phyML). (**b**) Phylogenetic analysis of HqiA showed that it is not related to any known AHL lactonases, otherwise clustering in the cysteine hydrolase group with other hypothetical (HYPO), isochorimatase-like (ISO) and *N*-carbamoylsarcosine amidase (NCAR)-like enzymes.

Comparison of the deduced protein sequences against the protein sequences available in the GenBank sequence database revealed that 31 out of 46 ORFs showed a high sequence homology with the class *Deltaproteobacteria* and among them, 25 ORFs equally distributed in the fosmid insert were related to sulphate-reducing bacteria (SRB) proteins (Table 1). The most represented genera related to the class *Deltaproteobacteria* were assigned to *Desulfobacula* (5 ORFs), *Desulfatibacillum* (3 ORFs), *Desulfobacterium* (4 ORFs), *Desulfosarcina* (2 ORFs), *Desulfomonile* (2 ORFs), *Desulforegula* (2 ORFs) and *Desulfocapsa* (2 ORFs). The other proteins identified on the fosmid were affiliated to *Gammaproteobacteria* (5 ORFs), *Betaproteobacteria* (2 ORFs), *Alphaproteobacteria* (1 ORF), *Clostridia* (1 ORF) and *Bacteroidetes* (2 ORFs) (Fig. 3). These affiliations were confirmed by phylogenetic analyses of the representative proteins of each genus based on maximum likelihood (phyML) (Fig. 4a). This approach confirmed the results obtained by BLAST, showing that the fosmid insert originated mainly from a *Deltaproteobacteria* genomic background. However, several ORFs (4, 5, 15, 27, 28, 29, 31, 35, 36, 38, 39, 40, 42, 43 and 46) related to other lineages, such as *Clostridia*, *Bacteroidetes*, *Alpha*-, *Beta*- or *Gammaproteobacteria*, were inserted in different locations between the *Deltaproteobacteria* related ORFs, suggesting that they were inherited by horizontal gene transfer.

**Table 1:** Phylogenetic analysis of each ORF in the fosmid f10/17.1H.

ORF	Length	Hypothetical function	Organism	Phylum/class
1	416	Hypothetical protein	Uncultured *Deltaproteobacteria*	*Deltaproteobacteria*
2	326	Thioredoxin	Uncultured *Deltaproteobacteria*	*Deltaproteobacteria*
3	2072	Two component system sensor histidine kinase, hybrid	*Desulfobacula toluolica*	*Deltaproteobacteria*
4	737	Hypothetical protein	*Uliginosibacterium gangwonense*	*Betaproteobacteria*
5	311	Nickel ABC transporter ATP-binding protein	*Enterobacter lignolyticus*	*Gammaproteobacteria*
6	533	Chemotaxis protein CheW	*Desulfatibacillum alkenivorans*	*Deltaproteobacteria*
7	1730	Fis family transcriptional regulator	*Desulfobacter postgatei*	*Deltaproteobacteria*
8	1199	Methyl-accepting chemotaxis sensory transducer	*Desulfobacula toluolica*	*Deltaproteobacteria*
9	359	Hypothetical protein	*Desulfovibrio alaskensis*	*Deltaproteobacteria*
10	419	Gliding motility protein	*Desulfosarcina* sp.	*Deltaproteobacteria*
11	680	CheY-like receiver, AAA-type ATPase and DNA-binding domain	*Desulfocapsa sulfexigens*	*Deltaproteobacteria*
12	743	Hypothetical protein	*Desulfosarcina* sp.	*Deltaproteobacteria*
13	371	Fis family transcriptional regulator	*Desulfonatronospira thiodismutans*	*Deltaproteobacteria*
14	1541	Chemotaxis protein CheA	*Desulfonauticus* sp.	*Deltaproteobacteria*
15	422	Fis family transcriptional regulator	*Pseudomonas fuscovaginae*	*Gammaproteobacteria*
16	524	CheY-like receiver, AAA-type ATPase and DNA-binding domain	*Desulfocapsa sulfexigens*	*Deltaproteobacteria*
17	416	Chemotaxis protein histidine kinase-like kinase	*Lawsonia intracellularis*	*Deltaproteobacteria*
18	512	Chemoreceptor glutamine deamidase CheD	*Desulfobacula toluolica*	*Deltaproteobacteria*
19	398	Histidine kinase	*Desulforegula conservatrix*	*Deltaproteobacteria*
20	2216	Chemotaxis protein A CheA	*Desulfobacula toluolica*	*Deltaproteobacteria*
21	482	Chemotaxis protein CheD	*Desulforegula conservatrix*	*Deltaproteobacteria*
22	428	Chemotaxis protein CheY	Uncultured *Desulfobacterium* sp.	*Deltaproteobacteria*
23	353	Hypothetical protein	*Desulfobacterium autotrophicum*	*Deltaproteobacteria*
24	1046	Chemotaxis response regulator protein-glutamate methylesterase	Uncultured *Desulfobacterium* sp.	*Deltaproteobacteria*
25	824	CheR-type MCP methyltransferase	*Desulfatibacillum alkenivorans*	*Deltaproteobacteria*
26	1046	L-threonine 3-dehydrogenase	*Desulfomonile tiedjei*	*Deltaproteobacteria*
27	1184	2-amino-3-ketobutyrate coenzyme A ligase	*Clostridium termitidis*	*Clostridia*
28	1232	NhaP-type Na^+^ (K^+^)/H^+^ antiporter	*Thioflavicoccus mobilis*	*Gammaproteobacteria*
29	725	Isochorismatase	*Hyphomonas jannaschiana*	*Alphaproteobacteria*
30	1271	4Fe-4S ferredoxin	*Desulfomonile tiedjei*	*Deltaproteobacteria*
31	839	GTP cyclohydrolase	*Pandoraea* sp.	*Betaproteobacteria*
32	428	Hypothetical protein	Uncultured *Deltaproteobacteria*	*Deltaproteobacteria*
33	1019	Phospholipid/glycerol acyltransferase	*Desulfatibacillum alkenivorans*	*Deltaproteobacteria*
34	785	Conserved hypothetical membrane protein	Uncultured *Deltaproteobacteria*	*Deltaproteobacteria*
35	275	Hypothetical protein	*Polaribacter* sp.	*Bacteroidetes*
36	647	Heme-binding protein	*Methylomonas methanica*	*Gammaproteobacteria*
37	347	Hypothetical protein	Uncultured *Deltaproteobacteria*	*Deltaproteobacteria*
38	1124	Uncharacterized ATPase	Uncultured bacteria	Unassigned
39	761	Hypothetical protein	*Pseudoalteromonas luteoviolacea*	*Gammaproteobacteria*
40	506	Hypothetical protein	Uncultured bacteria	Unassigned
41	866	Hypothetical protein	*Thermodesulfobium narugense*	*Deltaproteobacteria*
42	245	Unknown	*—*	Unassigned
43	1127	TonB-dependent siderophore receptor	*—*	Unassigned
44	302	Hypothetical protein	*Desulfobacula* sp.	*Deltaproteobacteria*
45	845	Unknown	Uncultured *Desulfobacterium* sp.	*Deltaproteobacteria*
46	989	Subtilisin-like serine protease	*Ignavibacterium album*	*Bacteroidetes*

The table presents the ORF number, the length in bp, the hypothetical function and the phylogenetic affiliation at both genus and phylum/class levels.

In term of function, a detailed analysis of the fosmid sequence revealed a cluster of ORFs homologous to genes encoding chemotactic signalling systems. Indeed, from ORF6 to ORF25 up to 13 genes related to chemotaxis and motility are represented, such as homologues of CheA (cytoplasmic histidine kinase), CheD (receptor-kinase coupling protein), CheR (methyltransferase), CheW (receptor-kinase coupling protein) and CheY (response regulator). The functionality of these genes was analysed using *E*. *coli* RP437 and two mutants (∆*cheA* or ∆*cheW*) unable to swarm. No complementation of the swarming phenotype was obtained after transformation of the mutants with f10/17.1H compared to the wild-type strain (data not shown). From ORF26 to ORF31, five ORFs presented homologies with putative enzymes, such as L-threonine-3-dehydrogenase (ORF26), 2-amino-3-ketobutyrate coenzyme A ligase (ORF27), isochorismatase (ORF29), a GTP cyclohydrolase (ORF31) and a phospholipid acyltransferase (ORF33). From ORF32 to 46, most of the ORFs were assigned as hypothetical proteins, except some ORFs such as ORF36 and ORF43 presenting homologies with proteins related to iron access. Notably, no homologues of the known AHL-degrading enzymes such as lactonases (AttM, AiiA, QlcA, QsdA) or acylases (QuiP, PvdQ) have been detected among the 46 ORFs carried by the fosmid (Table 1 and Fig. 3).

### Demonstration of the AHL degradation role conferred by the ORF29

A first step consisting in the cloning in pGEM-T of 5-kb fragments of the fosmid DNA insert was conducted to determine which region conferred AHL-degradation ability to *E*. *coli*. This first screening allowed the identification of a fragment containing from ORF26 to ORF31. Based on the sequence homology and the predicted enzymatic reaction associated to the ORF26, ORF27, ORF29, and ORF31, we selected and designed specific primers to clone the ORF29, which was predicted to encode an isochorismatase (237 aa). The isochorismatase corresponds to a large family of enzymes related to the cysteine hydrolases (CSHases) capable of cleaving ester and ether bonds. The ORF29 was first cloned in pGEM-T and then transferred to the low copy number plasmid pME6010 under the constitutive promoter pK. The AHL-degradation assays performed with pGEM-T or pME6010-based constructions revealed that ORF29 (named as *hqiA* gene) conferred to *E*. *coli* the ability to degrade all the AHLs tested (data not shown).

### HqiA: a new AHL-degradation enzyme type

A detailed analysis of the amino acid sequence highlighted that HqiA has no significant homology with the known AHL lactonase or acylase enzyme types. Indeed, HqiA appeared not to be related at all to the known AHL acylases (i.e., PvdQ, QuiP) (data not shown) and very distant from the known AHL lactonases (i.e., AiiA, AttM, QlcA or QsdA) (Fig. 4b). It also confirmed that the HqiA protein sequence was more related to that of the *Hyphomonas* genus than to those of other *Deltaproteobacteria* genera, although less related homologues can be found in the genome of sequenced strains of *Desulfobacterium*, *Desulfosarcina* and *Desulfocapsa*. This phylogenetic analysis also highlighted that HqiA clustered into the CSHase group with other α/β-hydrolase enzymes, and more especially within isochorimatase-like and *N*-carbamoylsarcosine amidase-like enzymes. Moreover, HqiA shows the conserved catalytic domains (D, K and C) of the CSHase family and presents a very different three-dimensional structure than the other AHL lactonases based on a Phyre2 analysis^30^ (see Supplementary Fig. S1).

### *hqiA* encodes for an AHL lactonase

**Figure 5:**
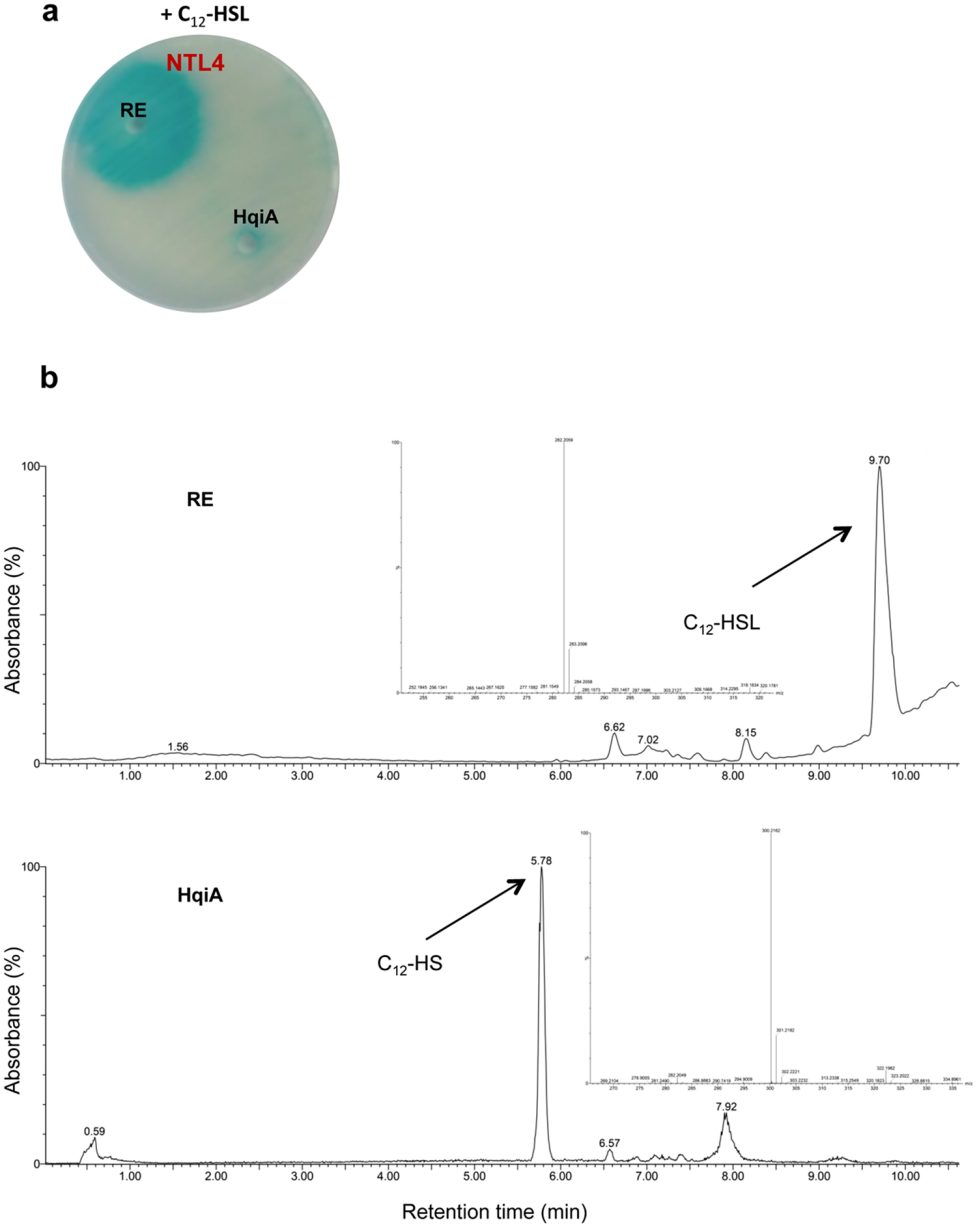
Functional characterization of purified HqiA. (**a**) Detection of the quorum-quenching activity against C_12_-HSL of the purified HqiA protein and the negative control RE (reaction buffer) using the biosensor *Agrobacterium tumefaciens* NTL4 (pZLR4). (**b**) HPLC/MS analysis of the C_12_-HSL degradation by HqiA. RE and HqiA sample chromatograms are shown. In the RE and HqiA samples, the main peaks masses are 282 and 300 which corresponds to the closed and open-ring form of the C_12_-HSL respectively, therefore indicating a lactonase activity.

To identify the function of the HqiA protein, *hqiA* was sub-cloned in the expression plasmid pMAL-c2TEV under the control of a Lac promotor to generate the fusion protein MBP-HqiA. The TEV protease-processed recombinant protein revealed a molecular weight of about 26 kD (see Supplementary Fig. S2). AHL-degrading activity of *hqiA* was confirmed against all the AHLs using a crude extract of induced *E*. *coli* carrying pMAL-c2TEV-ORF29, whilst no AHL degradation occurred with *E*. *coli* carrying the empty vector (data not shown). Furthermore, as revealed with biosensors, the degradation assays performed with the purified recombinant HqiA protein confirmed the AHL-degradation activity associated with the HqiA peptide (Fig. 5). To assign the enzymatic activity of the HqiA protein, the product of the enzymatic reaction between the purified recombinant protein and C_12_-HSL was identified by using both the lactone ring closure assay and HPLC/MS. Thus, after acidification of the supernatant with HCl and neutralisation of the pH, the C_12_-HSL-related signal was significantly recovered as evidenced using the *A*. *tumefaciens* biosensor (see Supplementary Fig. S3). Similar results were obtained with other AHLs (data not shown). The analysis by HPLC/MS of the main peak obtained after the C_12_-HSL degradation assay (HqiA) revealed a mass (M-) of 300, which corresponds to the open-ring form of the C_12_-HSL, while in the chromatogram corresponding to the control (RE) a molecular weight of 282, compatible with the closed form, was detected (Fig. 5).

### Quorum-quenching activity of HqiA against virulence of *Pectobacterium carotovorum* CECT 225^T^

**Figure 6:**
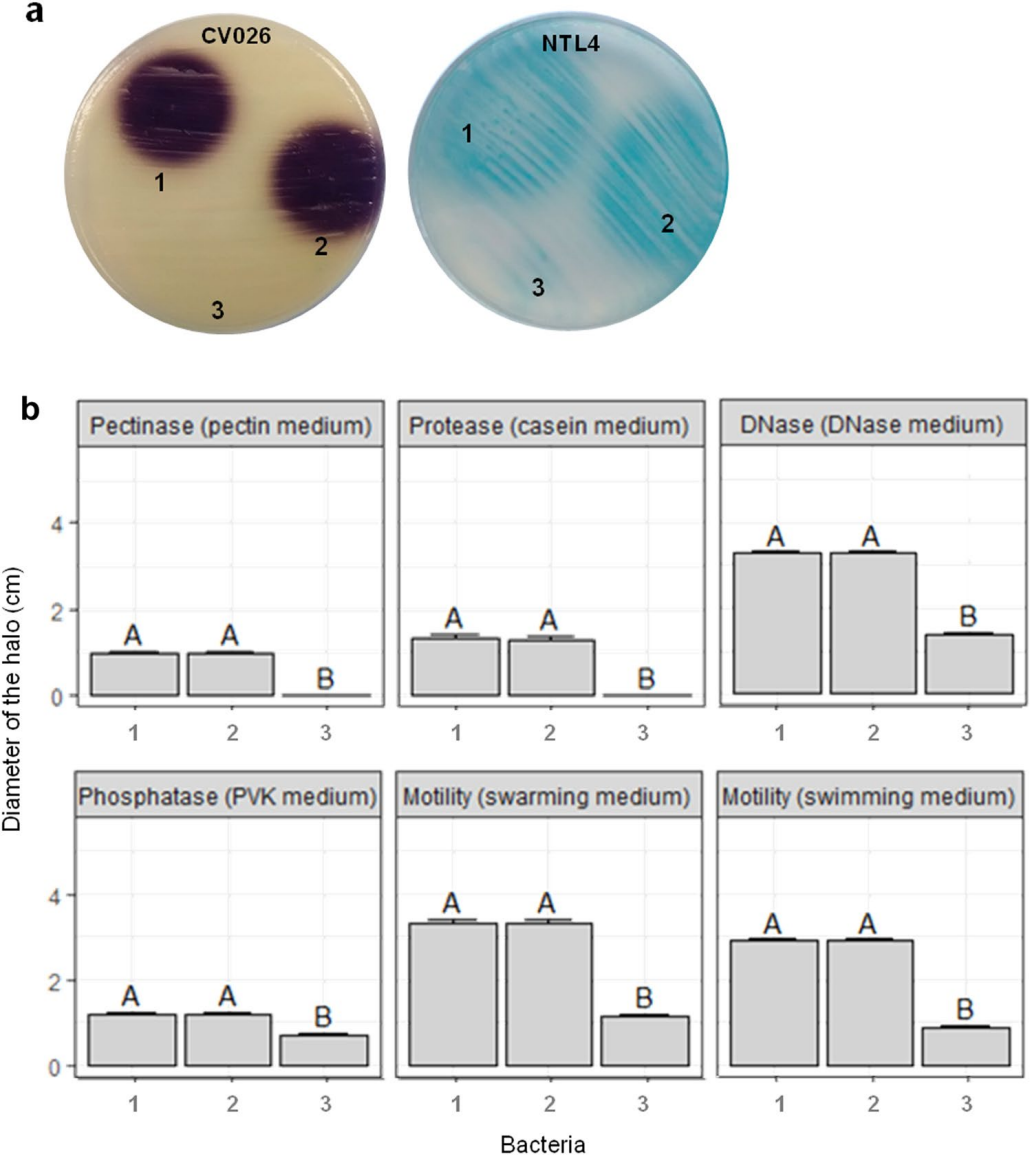
Quorum quenching of AHLs and QS-regulated functions in *Pectobacterium carotovorum*. (**a**) AHL detection using the biosensors *Chromobacterium violaceum* CV026 and *Agrobacterium tumefaciens* NTL4 (pZRL4). (**b**) Phenotypes tested in pectin, casein, DNase, PVK (alkaline phosphatase), swarming and swimming media. Error bars represent standard deviations. For each assay, the *P*. *carotovorum* strain without plasmid (1), with the empty plasmid (pME6010) (2) and the plasmid carrying *hqiA* (3) were compared. For the bioassays presented in panel b, different letters above the bars indicate that the values are significantly different according to a one-factor (treatment) ANOVA (P < 0.05) and the Tukey test.

**Figure 7:**
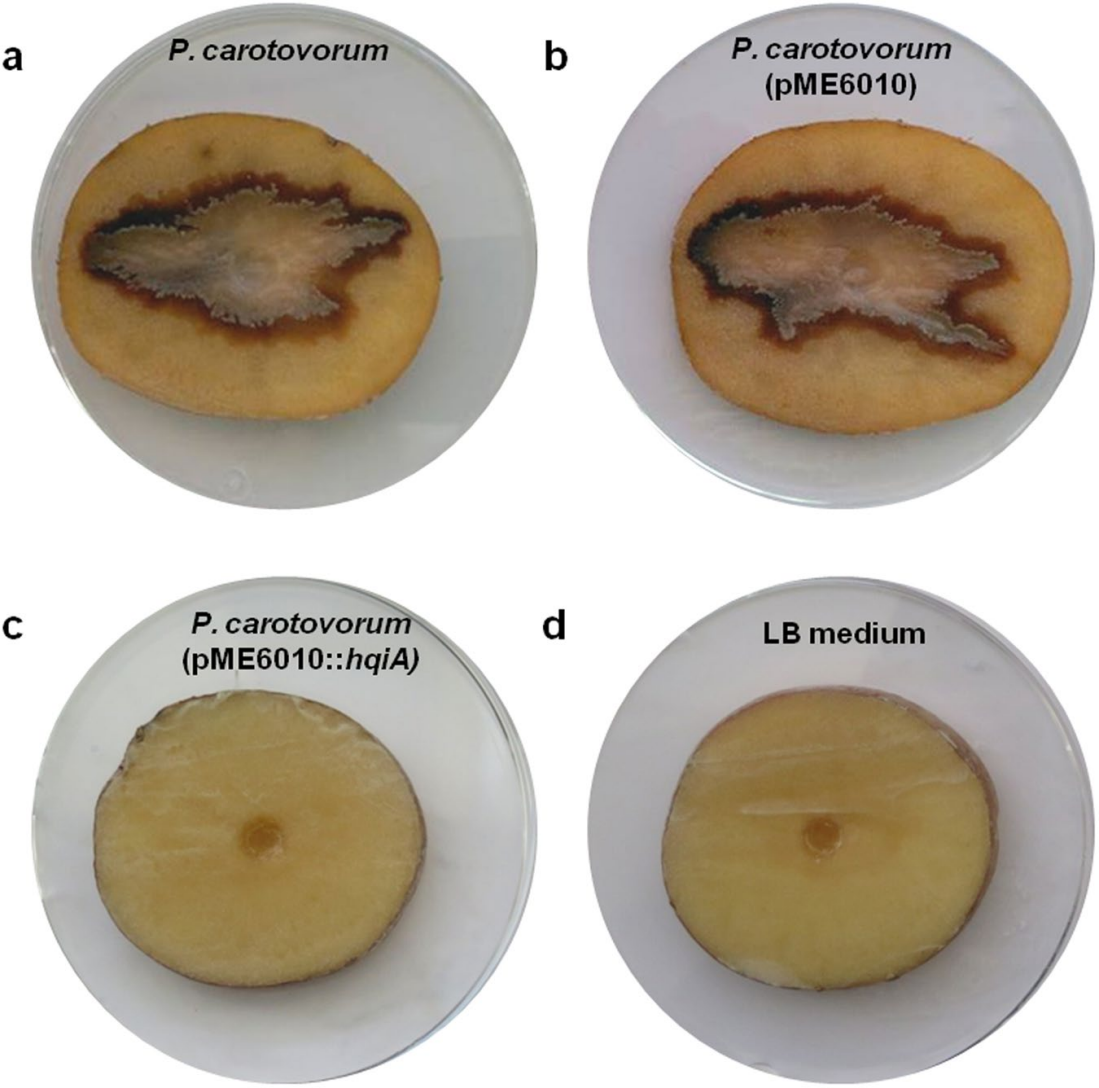
Virulence assay on potato tubers. Potato tuber slices inoculated with (**a**) *Pectobacterium carotovorum* without plasmid, (**b**) *P*. *carotovorum* (pME6010), (**c**) *P*. *carotovorum* expressing the *hqiA* gene and (**d**) cell-free LB medium.

In the plant pathogen *Pectobacterium carotovorum* subsp. *carotovorum* CECT 225^T^ virulence is related to maceration enzymes, such as protease or pectinase, swarming and swimming, among others, which are all regulated in a QS-dependent way and mediated by C_6_-HSL and 3-O-C_6_-HSL^31–33^. When the plasmid pME6010::*hqiA* was introduced into the pathogenic strain, the concentration of AHLs in the culture supernatant strongly decreased compared to the control performed with the empty plasmid. AHLs were no longer detected with *C*. *violaceum* CV026 and *A*. *tumefaciens* NTL4 (pZLR4) biosensors for the strain *P*. *carotovorum* carrying pME6010::*hqiA*, while they were clearly detected in the control conditions (Fig. 6a). The QQ ability of *hqiA* on *P*. *carotovorum* was then tested using the potato tuber assay. This bioassay highlighted that *P*. *carotovorum* carrying pME6010::*hqiA* was not able to cause soft rot symptoms (0% maceration on the surface of the potato slice), whilst the same bacterial strain carrying or not the empty plasmid caused a large maceration zone (39%) (Fig. 7). To test the impact of *hqiA* on the different QS regulated functions of *P*. *carotovorum*, cultures of *P*. *carotovorum* (pME6010::*hqiA*) and *P*. *carotovorum* carrying or not the empty plasmid (pME6010) were screened using different bioassays. This screening showed that compared to the bacterial strain carrying or not the empty plasmid, the introduction of *hqiA* into *P*. *carotovorum* abolished its pectinase, protease, DNase and alkaline phosphatase activities in our experimental conditions. In addition, swarming and swimming motilities were also blocked (Fig. 6b, see Supplementary Fig. S4). In all the bacterial cultures containing the plasmid with or without *hqiA*, cell counts were significantly similar showing that the expression of *hqiA* did not affect the growth of *P*. *carotovorum*.

## Discussion

During the last decades, QS and QQ have been described in many bacteria inhabiting different aquatic and terrestrial environments^4, 34^. On the other hand, studies on QS signal communication in extremophilic bacteria are very scarce, and especially those focusing on halophilic microorganisms^35, 36^. The first demonstration of the existence of QS signal communication systems in bacteria inhabiting hypersaline environments was done by Llamas *et al*.^10^. They also revealed that the ability to produce AHLs was conserved in the family *Halomonadaceae*, one of the most frequently detected halophilic bacteria in such an environment^11^. These results suggest that QS signal communication systems are widespread among halophilic bacteria and may participate in their fitness in extreme environments, but they give no information about the other uncultivated bacterial taxa. Until very recently, most of our knowledge on QS and QQ relied only upon cultivation-based approaches, ignoring a large part of the microbial diversity and the functional potential of the bacterial communities occurring in complex and extreme environments^37, 38^. The first cultivation-dependent study focusing on the relative distribution of the bacteria from the soil capable of producing or degrading AHLs showed that 8–19% of these bacteria had the capability to produce AHLs, whilst 2–3% had the capability to degrade AHLs^39^. The proportion of potential AHL-producing bacteria was shown to reach 13% when considering the proteobacterial genomes available in the international sequence database NCBI, but these genes appeared far less frequently in non-proteobacterial genomes^40^. The metagenomic approaches based on shotgun sequencing or fosmid libraries gave very different pictures. Indeed, these approaches revealed lower frequencies but higher diversity of AHL-producing or degrading bacteria in the different environments analysed^29, 41, 42^. Considering only QQ homologues in various aquatic and terrestrial metagenomes, Romero *et al*.^29^ showed that the frequency of QQ homologues varied from 0.05 to 0.4 homolog per Mbp. However, QS and QQ genes are known to conserve a low sequence homology between them, consequently many homologues identified *in silico* in genomic and metagenomic data are non-functional^29, 43^. In this context, the use of fosmid libraries can be a good alternative as it permits the testing of clones for their ability to degrade or produce AHLs. New AHL-producing or degrading genes have been identified by this method^9, 28, 42, 44, 45^, but the frequency of obtaining positive clones remains low and varied from 4.82 × 10^−4^ to 3.3 × 10^−3^ per Mbp^9, 42^. In our metagenomic library generated from a hypersaline soil, a single clone was obtained after the screening of 250,000 clones, corresponding to an estimated frequency of 1 × 10^−4^ positive clone per Mbp. On the contrary, no genes related to the production of QS signal molecules have been detected. As AHL-producing bacteria have been evidenced in the same experimental site^10, 11^, we cannot exclude biased and insufficient expression of *E*. *coli* of some genes in fosmid-based metagenomic libraries^46^ or limited sensitivity of the screening strategy used.

The chemical characterization of the activity encoded by the *hqiA* gene by HPLC/MS highlighted that it encoded a lactonase activity characterized by a broad range of degradation of unsubstituted, oxo- and hydroxyl-substituted AHLs. To date, various types of prototypic AHL lactonases have been evidenced, such as the AiiA, AiiB, AidH, AttM, AhlK, QsdA or QlcA^22, 23, 42, 47–51^. These enzymes mostly identified by cultivation-dependent approaches can be clustered in two distinct groups: i) the Zn-metallohydrolases encompassing AiiA-like and phosphotriesterase (PTE)-like enzymes and ii) the α/β-hydrolases with only one characterized member (AidH). These enzyme types present specific functional motifs, such as HXDH-H-D for AiiA, AiiB, AttM, AhlK and QlcA, G-VL-HEH for QsdA or G-S-GG for AidH that permit their *in silico* identification. Notably, the only AiiA-like enzyme obtained by a fosmid screening (QlcA) was proposed as a new prototypic AHL lactonase based on phylogenetic analyses^42^. In our study, HqiA does not contain the structural motives typically found in the other known lactonases, such as AiiA, AttM, AhlK, QlcA, QsdA or AidH, suggesting that it represents a new family of AHL lactonases not related to the Zn-metallohydrolase (AiiA-like and PTE-like) enzymes. Moreover, a comparison of the three-dimensional structure of the different AHL lactonases and of HqiA confirmed that they did not show a similar structure. Notably, an *in silico* analysis revealed that HqiA presents strong homology with other types of enzymes found in the α/β-hydrolase enzyme family but not related to AidH. Indeed, HqiA appeared to present homology with enzymes grouped in the cysteine hydrolase (CHase) group. Members of this enzyme group have been shown to cleave ether, ester and amide bonds. The higher sequence homologies of HqiA were obtained with isochorismatase-like and *N*-carbamoylsarcosine amidase-like enzymes. Diverse biological functions have been assigned to bacterial isochorismatases, but never related to AHL degradation. As an example, HqiA presents a strong homology with PhzD, which is described as an isochorismatase involved in the phenazine biosynthesis in *Pseudomonas aeruginosa* PAO1^52^. Interestingly, chemical assays identified both AHL-acylase and AHL-lactonase activities in *P*. *aeruginosa*. To date, three AHL acylase genes have been identified in *P*. *aeruginosa* PAO1, but a lactonase gene has not yet been identified^34, 53–55^. Even if sequence homology is not always a proof of function, our future aim is to conduct the functional characterization of PhzD, which would perhaps expand the list of QQ enzymes in *P*. *aeruginosa* PAO1 with an AHL lactonase as well as it would help to better understand the regulation of QS in this pathogenic bacterial strain.

Regarding the taxonomic origin of the environmental DNA cloned in fosmid f10/17.1H, *in silico* analysis (BLAST search and phylogenetic analyses) revealed that 31 out of the 46 ORFs showed high sequence homology with *Deltaproteobacteria*, suggesting that the environmental DNA might originate from this bacterial class. Additionally, 25 out of these 31 ORFs were related to sulphate-reducing bacteria (SRB), a group of microorganisms ubiquitous in hypersaline environments, where sulphate is a major inorganic compound^56, 57^. Interestingly, a large portion of the Deltaproteobacterial ORFs (n = 13) presented strong homology with chemotaxis and motility systems. These genes probably provide a competitive advantage to bacteria in the colonization of hypersaline soil. The organization of *che* genes in large clusters has been evidenced in other bacterial taxa, such as in *Sinorhizobium*, *Rhizobium* and *Azospirillum* ^58, 59^. Interestingly, chemotaxis was shown to be regulated by an AHL-based quorum sensing system in *Sinorhizobium meliloti*, but in our case we do not have evidence of such a link^60^. Besides the ORFs belonging to *Deltaproteobacteria*, our phylogenetic analysis revealed that many ORFs assigned to other phylogenetic groups (Al*pha-*, *Beta-* and *Gammaproteobacteria*, *Clostridia* and *Bacteroidetes*) were inserted between Deltaproteobacterial ORFs. Such gene organization without notable change of G + C% and the absence of evident mobile elements suggest that horizontal gene transfer (HGT) occurred. Horizontal gene transfer is described as a universal mechanism used by bacteria to acquire new functions that allow them to adapt to environments with different selective pressures. The presence of HGT events has already been reported in other hypersaline environments, suggesting that they also occur in the hypersaline soil of Rambla Salada^61–63^. The ORF29 (*hqiA*) encoding the AHL lactonase-like enzyme was assigned as the unique Alphaproteobacterial ORF of the cloned DNA, with a strong homology with homologous genes annotated as isochorismatases in the genomes of bacterial strains belonging to the *Hyphomonas* genus. Notably, homologues are also present as single copy in the genomes of *Desulfobacula toluolica* and *D*. *phenolica* but without evidence of HGT. In all the cases, the biological function of these other homologous genes has not yet been experimentally tested. However, the presence of inherited genes by HGT in the *Deltaproteobacteria* context suggests that they may confer a selective advantage. Recent observations extend the role of QQ enzymes to the detoxification of signalling molecules. Interestingly, an *in silico* analysis of the genome of a strongly related *Deltaproteobacteria* strain (*Desulfatibacillum alkenivorans* AK-01) revealed the presence of an annotated *aiiA*-like gene presenting high homology only with *Firmicutes* members. The identification of this other potential case of horizontal transfer of an AHL lactonase gene suggests that HGT events occur regularly between *Deltaproteobacteria* and other bacterial taxa and that QQ genes may be exchanged.

Although the real physiological role of the AHL-degrading enzymes remains to be determined^34^, such enzymes appear as a valuable alternative to chemicals and antibiotics used to treat diseases. Our QQ experiments showed that HqiA not only significantly degraded a wide range of AHLs, but that also interfered with the production of AHL molecules in the plant pathogenic bacterium *P*. *carotovorum* subsp. *carotovorum*. Moreover, we revealed that the expression of several QS-regulated phenotypes, such as maceration enzymes or motility were inhibited or strongly perturbed, thus confirming previous findings^22, 47, 64^. The QQ activity of HqiA was also verified in the AHL-producing bacteria *Agrobacterium fabrum* C58^2^, *Halomonas anticariensis* FP35^T10^ and *Vibrio coralliilyticus* VibC-Oc-193 (unpublished data). In each case, the introduction of a plasmid carrying *hqiA* allowed the degradation of the AHLs (data not shown). Although the QQ results obtained with HqiA in *P*. *carotovorum* are similar to those obtained using AiiA, AttM, AidH, QlcA or QsdA, they open up new perspectives in terms of diversity of enzymes^22, 47, 65^. Indeed, due to variations of codon usage or the problems of expression in other genomic backgrounds, the use of very different AHL-degrading enzyme types may be a good alternative to effectively quench bacterial pathogens.

To sum up, our study represents the first fosmid-based screening of a hypersaline soil metagenome to search for genes involved in QS and QQ. In this way, we were able to a new class of AHL-degradation-enzyme encoding gene (*hqiA*) not previously related to the known prototypes. Further studies will be necessary to demonstrate the biological function of HqiA, its substrate specificity and its conservation among bacterial genomes.

## Experimental procedures

### Sampling site

The soil sample considered in this study was collected in November 2007 in Finca La Salina (Rambla Salada, Murcia, Spain) (38°07′34.44″N, 1°07′11.13″W). This soil is considered as hypersaline and its physicochemical characteristics were previously determined^66^.

### Bacterial strains, media and growth conditions

*Escherichia coli* strains were grown in Luria-Bertani (LB) medium. *E*. *coli* RP437, its related chemotaxis mutants [RP9335 (RP437∆*cheA*) and UU2871 (RP437∆*cheW*)] and their transformants were grown in T-broth^67, 68^, they were tested for their swarming ability on T-broth 0.2% (w/v) agar. *Chromobacterium violaceum* CV026 and VIR07 biosensor strains and *Pectobacterium carotovorum* subsp. *carotovorum* CECT 225^T^ were grown in LB^69, 70^. *Agrobacterium tumefaciens* NTL4 (pZLR4) was cultured in AB medium supplemented with 80 μg ml^−1^ of 5-bromo-4-chloro-3-indolyl-β-D-galactopyranoside (X-gal) when needed^71^. All strains were grown at 28 °C.

When required, antibiotics where used at the following final concentrations: chloramphenicol (Cm) 12.5 µg ml^−1^, ampicillin (Amp) 50 µg ml^−1^, kanamycin (Km) 50 µg ml^−1^, streptomycin (Sm) 100 µg ml^−1^, gentamicin (Gm) 50 µg ml^−1^ and tetracycline (Tc) 10 µg ml^−1^.

All the AHLs used (Sigma®) [C_4_-HSL (*N*-butyryl-DL-homoserine lactone), C_6_-HSL (*N*-hexanoyl-DL-homoserine lactone), 3-O-C_6_-HSL (*N*-3-oxo-hexanoyl-DL-homoserinelactone), C_8_-HSL (*N*-octanoyl-DL-homoserine lactone), 3-O-C_8_-HSL (*N*-3-oxo-octanoyl-DL-homoserinelactone), C_10_-HSL (*N*-decanoyl-DL-homoserine lactone), 3-OH-C_10_-HSL (*N*-3-hydroxydecanoyl-DL-homoserine lactone), C_12_-HSL (*N*-dodecanoyl-DL-homoserine-lactone), 3-O-C_12_-HSL (*N*-3-oxo-dodecanoyl-DL-homoserinelactone) and C_14_-HSL (*N*-tetradecanoyl-DL-homoserine lactone)] were used at final concentration of 25 µM.

### DNA extraction and preparation of the metagenomic library

Total DNA was extracted from 10 g of soil sample using the MoBio PowerMax™ Soil DNA Isolation Kit (MoBio Laboratories) following the protocol provided by the manufacturer. To generate DNA compatible with the construction of the metagenomic library, DNA was sheared manually by pipetting several times and separated using a sucrose gradient. Briefly, 100 µg of genomic DNA were loaded in 17 ml tubes and separated according to their molecular size through a 5 to 40% (w/v) sucrose gradient. This separation was done at 15,000 × *g* at 10 °C for 20 h with an L-80 XP Optima ultracentrifuge (Beckman Coulter) associated to a swinging bucket rotor SW28.1 Ti (Beckman Coulter). After the ultracentrifugation, fractions of 200 µl were collected and the fractions of interest (~40 kb) were pooled and used for the construction of the metagenomic library using the CopyControl Fosmid Library Production Kit (pCC1FOS vector; Epicentre).

### Screening and selection of clones with quorum-quenching activity

All the experiments were conducted at pH 7 to prevent the lactonolysis of the AHLs^72^. The metagenomic library was diluted in a total volume of 500 ml of LB Cm to obtain a final concentration of 250 clones per ml. 200 µl of this suspension were distributed per well in 96-well microtiter plates (~50 clones per well) and incubated for 24 h at 37 °C following the procedure described by Uroz and Oger^73^. After this incubation time, 100 µl of each well were transferred in new 96-well microtiter plates containing fresh LB medium supplemented with C_6_-HSL and incubated at 28 °C for 24 h. Then the C_6_-HSL degradation was tested in microtiters using the biosensor *C*. *violaceum* CV026^74^. LB cell-free medium supplemented with C_6_-HSL was used as a negative control. Positive wells without violacein production were retained as wells containing clones with potential QQ activity for further analysis and fosmids were purified using the Midi Prep Purification kit (Qiagen).

To determine the AHL-degradation activity of the selected fosmids, 500 µl of an overnight culture of *E*. *coli* S17 λ *pir* carrying the fosmid were incubated at 28 °C for 24 h in LB medium supplemented with 25 µM of each of the synthetic AHLs. LB cell-free medium and *E*. *coli* S17 λ *pir* supplemented with the same concentration of AHLs were incubated at 28 °C for 24 h as negative controls. The remaining AHLs in each overnight culture were detected on AB or LB agar plates overlaid with the appropriate biosensor strains to check for the appearance of a coloured halo around each spot^74^.

### Confirmation of AHL-degradation activity by TLC and HPLC/MS

To exclude a QQ ability linked to the production of an inhibitor, supernatants of cultures were analysed by thin-layer chromatography (TLC) and high-performance liquid chromatography/mass spectrometry (HPLC/MS). For the TLC analyses, 10 µl of supernatants of degradation assays performed in LB supplemented with C_6_-HSL or C_12_-HSL were spotted onto TLC plates (Partisil KC18 Whatman 20 × 20 cm plates) and developed with CH_3_OH:H_2_0 60:40 (v/v) as mobile phase. After migration, the plates were air-dried and overlaid with top agar containing the appropriate biosensor and incubated at 28 °C for 24 h^10, 75^.

For the HPLC/MS analysis and quantification, 500 µl of supernatants of degradation assays of C_6_-HSL and C_12_-HSL and non-inoculated media (negative control) were extracted twice with an equal volume of dichloromethane, evaporated and resuspended in 400 µl of acetonitrile^26^. Analyses were carried out with an Agilent HPLC 1100 chromatograph, equipped with a C8 precolumn (2.1 × 12.5 mm, 5 μm) and a Zorbax Eclipse XDB-C18 column (2.1 × 150 mm, 5 μm) maintained at 45 °C. The mobile phase was built by HCOOH:H_2_O 1:1000 (v/v) and HCOOH:C_2_H_3_N 1:1000 (v/v)^76^. Mass spectrometry experiments were conducted on an API 4000 triple-quadrupole mass spectrometer (Applied Biosystem) equipped with a Turbolon source using the positive ion electrospray and multiple reaction monitoring (MRM) mode. The MRM signals were used to generate relative quantification information by comparison with a calibration curve constructed for molecular ion abundance, using each of the appropriate AHL synthetic standards^77^. The effects of the AHL chain length (C_6_-HSL vs C_12_-HSL) and of the treatment (*E*. *coli* with an empty fosmid or with the fosmid containing *hqiA*) on the AHL-degradation efficiency were determined by analysis of variance using a two-factor (ANOVA) test (P < 0.05) and a Tukey test using R.

### Sequencing of the AHL-degrading f10/17.1H fosmid, annotation and phylogenetic analyses

Sequencing of the fosmid f1017.1H was performed on a 454 GS Junior system as recommended by the manufacturer and according to the rapid library production and sequencing method protocols (Roche). Briefly, fosmid f1017.1H was diluted in TE buffer (10 mM Tris-Cl, pH 7.5, 1 mM EDTA) to reach a concentration of 5 ng µl^−1^ and this suspension was physically fractionated by nebulisation to generate 450 bp fragments. After ligation of the adapters, emulsion PCR was performed using the emPCR amplification kit (Lib-L, Roche). The run of pyrosequencing yielded 50,457 reads. These sequences were used to generate a *de novo* assembly with the Roche 454 sequencing assembly software Newbler. This assembly resulted in one contig of 50,457 bp, corresponding to 8,139 bp of the pCC1FOS fosmid and to 42,318 bp of metagenomic insert. ORFs in this insert were predicted by using the RAST and GeneMark applications^78, 79^. After gene prediction, nucleotide and aminoacid sequence analyses were made using BLAST^80^. G + C content was calculated using the web tool GC-Profile^81^. For each protein encoded by the fosmid f1017.1H, homologous protein sequences were retrieved from NCBI after identification by BLAST X^80^. Multiple sequence alignments and phylogenetic trees were constructed using Seaview^82^. Briefly, alignments were carried out with ClustalO^83^. Maximum likelihood (ML) bootstrap analyses were carried out with 1000 replicates. Phylogenetic trees were constructed with PhyML using the LG model, the NNI (nearest-neighbor interchange) and BioNJ options to improve likelihood and tree topology.

The fosmid sequence was deposited on SRA trace archive system under bioproject number PRJNA326320 and biosample number SAMN05276011.

### Cloning of ORF29 and confirmation of AHL-degradation activity

To identify the gene coding for the QQ ability in the fosmid f10/17.1H, subcloning of the metagenomic insert DNA was carried out. A PCR-based strategy was applied to test the potential ORFs involved. First, 5-kb PCR fragments of the fosmid insert were cloned and tested for their ability to degrade AHLs. After this first step and on the basis of its sequence homology with a hydrolytic enzyme, ORF29 was selected. It was amplified using the specific primers ORF29 forward 5′-CATTTCAGCAAAACCATGGAGGTGA-3′ and ORF29 reverse 5′-TCAAAACGAGCTCACGGTGCAAT-3′ that include (underlined) a *Nco*I and *Sac*I restriction sites respectively. Then it was cloned in pGEM-T and transferred to *E*. *coli* DH5α, where the QQ ability of the construction was tested in LB supplemented with C_6_-HSL as described previously. After verification of the QQ phenotype, ORF29 was digested with *Nco*I and *Sac*I enzymes and transferred into the pME6010 broad-host-range cloning vector^84^ for construction of pME6010::*hqiA* (for hypersaline quorum-quenching isochorismatase). Then, it was transformed into *E*. *coli* S17 λ *pir*. The QQ activity of this construction was tested in LB supplemented with different AHLs as previously described in experimental procedures. *E*. *coli* S17 λ *pir* harbouring the empty plasmid pME6010 was used as negative control.

### Cloning, expression and purification of the HqiA protein

To allow protein production, the *hqiA* gene was amplified using the primer pair *hqiA* forward 5′-GGATCCATGAGTGAAATCAGCTTGGCG-3′ and *hqiA* reverse 5′-AAGCTTTTACGATCCTTCGGGTAAAGAAC-3′ that include (underlined) a *BamH*I and *Hind*III restriction sites to facilitate the cloning in a modified pMAL-c2X expression vector (Cm^R^) (New England Biolabs) where the factor Xa cleavage site has been substituted with a TEV protease cleavage site. The resulting plasmid was termed pMAL-c2TEV-ORF29 and the plasmid sequence was confirmed by automatic sequencing with universal primers.

*E*. *coli* BL21(DE3)pLysS was transformed with pMAL-c2TEV-ORF29 and cultured at 37 °C until OD_600nm_ 0.5. Then protein expression was induced with 1 mM isopropyl β-D-1-thiogalactopyranoside (IPTG) for 6 h at 37 °C. After centrifugation, cell pellets were resuspended in a cell-lysis buffer (CLB) (20 mM Tris-HCl, 50 mM NaCl, 1 mM EDTA, pH 7.5) and sonicated. The soluble fraction was clarified by centrifugation at 10,000 × *g* for 20 min. Supernatants were applied to MBPTrap HP columns (GE Healthcare) and washed with CLB until the protein content in the wash was negligible. Then, the recombinant protein in the column was eluted with CLB supplemented with 10 mM maltose. The eluted fractions were pooled and digested overnight at 4 °C in the same buffer with 1 mg of recombinant TEV protease for each 25 mg of protein^85^. The digested samples were loaded in DEAE Sephacel columns (GE Healthcare) and the protein was eluted with a NaCl linear gradient (100 to 500 mM NaCl). The eluted fractions were pooled and stored at −20 °C. Protein concentration was assayed using the Bradford method^86^. The potential three-dimensional structure of the purified protein was calculated using the software Phyre2, a web portal for protein modeling, prediction and analysis based on aminoacid sequences^30^.

### Characterization of the AHL-degrading activity of purified HqiA

AHL-degradation activity of the purified HqiA was tested in reaction buffer (RE) (200 mM Na phosphate, pH 6.5) containing 16 µg ml^−1^ of HqiA protein and 25 µM of each of the synthetic AHLs. After incubation at 28 °C for 4 h, the remaining AHLs were detected on agar plates overlaid with *A*. *tumefaciens* NTL4 (pZLR4) as previously described above. To identify the type of QQ enzyme, the degradation products of the QQ reaction of HqiA were analyzed by HPLC/MS and by the lactone ring closure assay after acidification^72, 74^. HPLC/MS was conducted on a Waters Acquity UPLC equipped with an UPLCr BEH C18 reverse-phase column (100 × 2.1 mm, 1.7 µm) coupled to an ESI-MS detector (Waters Synapt G2). Samples were injected and eluted with NH_4_OH:H_2_O 1:1000 (v/v) and NH_4_OH:C_2_H_3_N 1:1000 (v/v). Mass spectrometry experiments were recorded with ES- Polarity. The lactone ring closure assay consisted of the acidification to pH 2 the reaction after incubation of HqiA with the different AHLs tested. This condition allows the lactone ring to restructure itself in the case that it had previously been opened by a lactonase. After neutralizing the pH, remaining AHLs were detected were detected on AB or LB agar plates overlaid with the appropriate biosensor.

### Pathogenicity test and extracellular enzymatic assays

The ability of *hqiA* to interfere with the expression of QS-regulated functions was tested in a heterologous expression system. The construction pME6010::*hqiA* and the empty plasmid pME6010 (Tc^R^), which was used as negative control, were transferred into the plant pathogen *Pectobacterium carotovorum* subsp. *carotovorum* CECT 225^T^ by electroporation^87^. To confirm that *P*. *carotovorum* subsp. *carotovorum* contained pME6010::*hqiA*, PCR amplification was carried out using specific primers *hqiA*-T forward 5′-ATGAGTGAAATCACGTTGGC-3′ and *hqiA*-T reverse 5′-CTTTACCCGAAGGATCGTAA-3′. To determine the impact of the expression of *hqiA* on AHL-production by the pathogenic strain, an agar-diffusion assay was carried out using the biosensors *C*. *violaceum* CV026 and *A*. *tumefaciens* NTL4 (pZLR4). Briefly, 20-µl of overnight cultures (OD_600nm_ 2.7) of the strain CECT 225^T^ carrying or not the plasmid pME6010 or pME6010::*hqiA* were spotted in LB and AB agar plates previously overlayed with the biosensor strains. To check that the expression of the *hqiA* gene did not affect the growth of this bacterial strain, serial dilutions of overnight cultures (OD_600nm_ 2.7) of the strain CECT 225^T^ carrying or not the different constructions were conducted and cell counts of each culture were performed on LB agar plates supplemented or not with Tc.

To evaluate the effect of the AHL-degradation of *hqiA* upon some of the QS-regulated virulence factors produced by the strain *P*. *carotovorum*, 20-µl of overnight cultures (OD_600nm_ 2.7) of the strain CECT 225^T^ carrying or not the plasmid pME6010 or pME6010::*hqiA* were spotted on different enzymatic media. Proteolytic activity was determined in casein medium^88^. DNase activity was determined in DNase agar medium^89^. The swimming motility test was carried out in LB 0.3% (w/v) agar^90^. The swarming motility test was carried out in LB 0.5% (w/v) agar^91^. Pectinase activity was carried out in pectin medium^92^. Alkaline phosphatase activity was determined in PVK medium^93^. Five replicates were used for each condition. In all these enzymatic media the result was obtained by measuring haloes around the spotted area, except for the motility tests where growth due to the migration of cells away from the inoculation site was measured. The diameter of haloes in each medium was measured and statistical analyses were conducted using an ANOVA test (P < 0.05) and a Tukey test using R.

The ability of *hqi*A to interfere with the pathogenicity of the strain CECT 225^T^ was also tested in a potato tuber assay^12^. Briefly, the strain CECT 225^T^ carrying or not the plasmid pME6010 or pME6010::*hqiA* were grown at 28 °C for 24 h (OD_600nm_ 2.7). Potato tuber slices (*Solanum tuberosum*) were surface sterilized using 10% (v/v) sodium hypochlorite during 15 min, rinsed with sterile distilled water, cut in slices and placed in Petri dishes with wet filter paper to keep them moist. 20 µl of each culture was inoculated on the surfaces of the potato slice and incubated at 28 °C for 48 h. LB cell-free medium was also inoculated on potatoes as a negative control. Five replicates were used for each condition. Symptoms were recorded after a 2-day incubation by visual inspection of the maceration zones. Appearance or not of maceration zones was statistically analysed using an ANOVA test (P < 0.05) and a Tukey test using R. The spatial extent of the maceration zone was estimated by image analysis using ImageJ software^94^ as a percentage of the potato slice surface area.

## Electronic supplementary material

Supplementary Material
