# Supplementary material for: HqiA, a novel quorum-quenching enzyme which expands the AHL lactonase family
Source: Sci Rep. 2017 Apr 19;7:943. doi: 10.1038/s41598-017-01176-7 (PMC5430456; doi:10.1038/s41598-017-01176-7)
Supplement: Supplementary file 1 — Supplementary Material [file 41598_2017_1176_MOESM1_ESM.pdf]

Supplementary material

HqiA, a novel quorum-quenching enzyme which expands the AHL lactonase family

Marta Torres[a,b], Stéphane Uroz[c], Rafael Salto[d], Laure Fauchery[c], Emilia Quesada[a,b], Inmaculada Llamas[a,b]*

[a]Department of Microbiology, Faculty of Pharmacy, University of Granada, Granada, Spain.

[b]Institute of Biotechnology, Biomedical Research Center (CIBM), University of Granada, Granada, Spain.

[c]UMR 1136 INRA-Université de Lorraine Interactions Arbres-Microorganismes, Centre INRA de Nancy, Champenoux, France.

[d]Department of Biochemistry and Molecular Biology II, Faculty of Pharmacy, University of Granada, Granada, Spain.

*Author for correspondence: Inmaculada Llamas. Department of Microbiology, Faculty of Pharmacy, Campus de la Cartuja s/n, Granada, Spain. 958243871. illamas@ugr.es

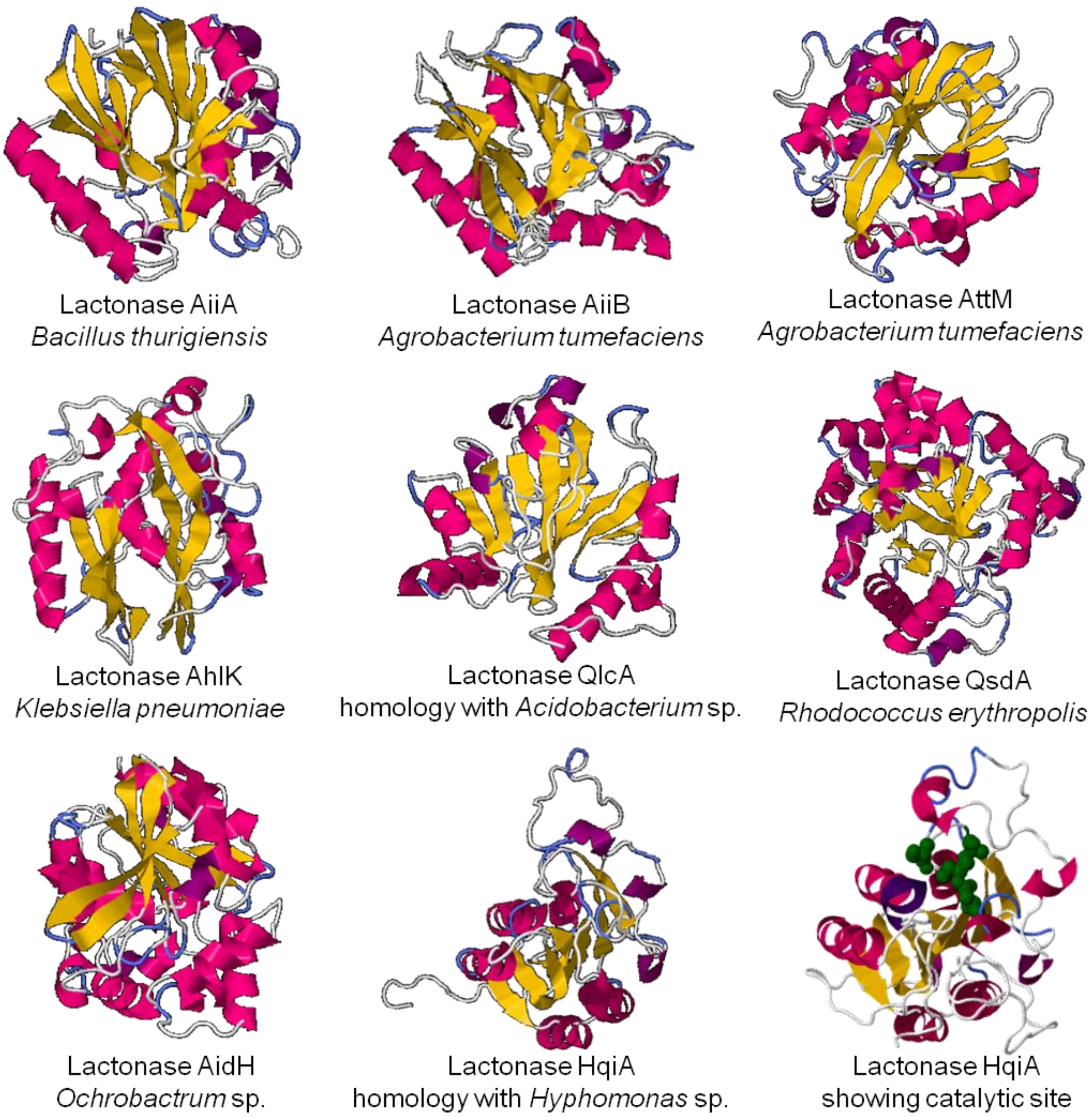

Supplementary Figure S1. Protein structure of prototypic lactonases and HqiA. HqiA showing the conserved catalytic domains D38, K144 and C147 (highlighted in green) of the CSHase family.

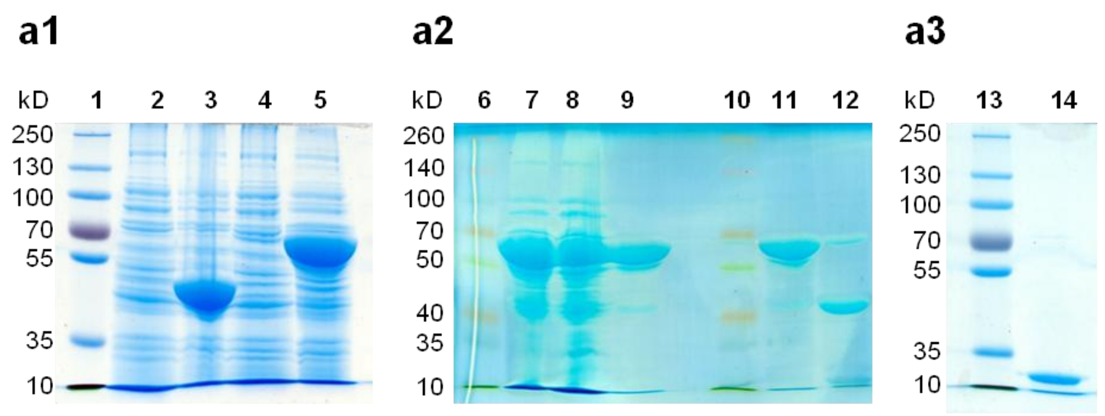

Supplementary Figure S2. HqiA protein expression and purification. Protein electrophoresis of MBP-HqiA expression (a1). Lane 1: protein molecular weight marker, lanes 2 and 3: non-induced and induced extracts from empty vector transformed bacteria (coding for a 42.2 kD protein), lanes 4 and 5: non-induced and induced extracts of pMAL-c2TEV-ORF29 transformed bacteria (coding for a 68.7 kD fusion protein). MBP-HqiA purification and proteolytic processing (a2). Lanes 6 and 10: protein molecular weight marker, lane 7: crude extracts, lane 8: soluble extracts, lanes 9 and 11: affinity chromatography fractions, lane 12: TEV protease processed fusion protein. Ion exchange chromatography (a3). Lane 13: protein molecular weight marker, lane 14: ion exchange chromatography eluted sample.

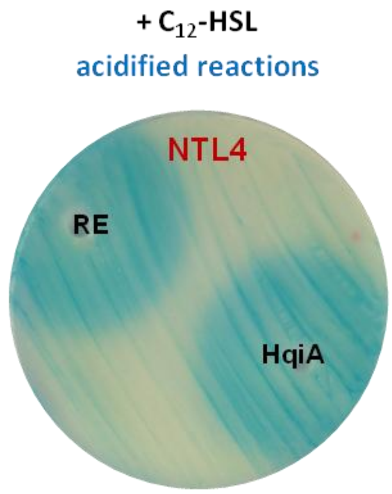

Supplementary Figure S3. **Lactone ring closure assay.** Detection of AHLs in the reaction buffer (RE) and HqiA protein with C_{12}-HSL after acidification using the biosensor *Agrobacterium tumefaciens* NTL4 (pZLR4).

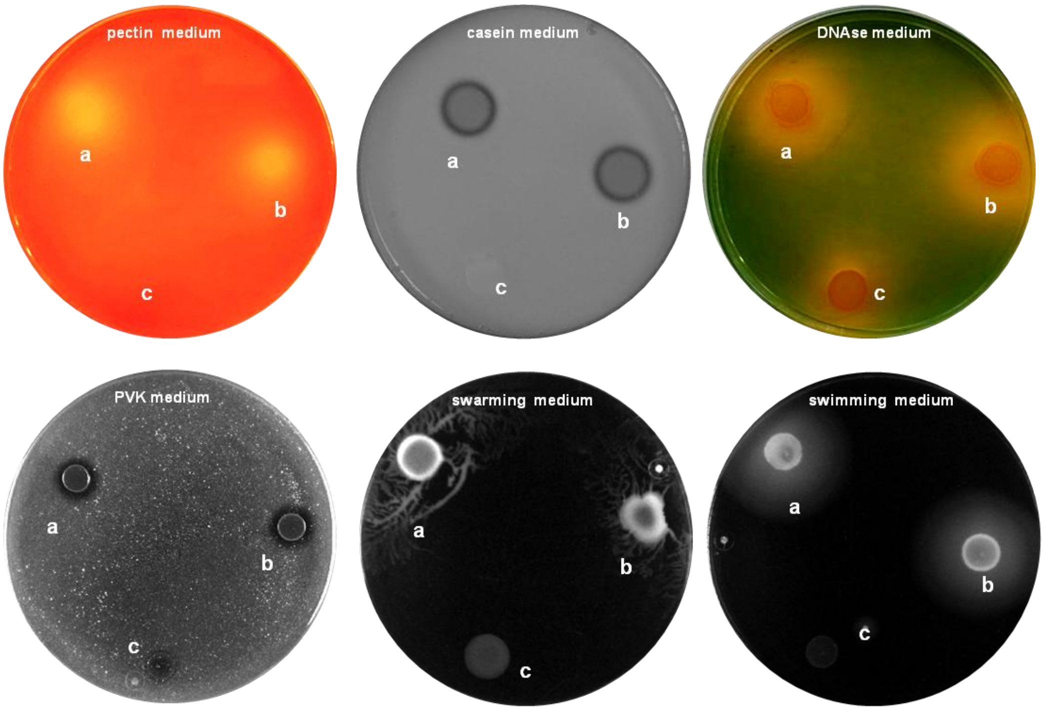

Supplementary Figure S4. Influence of *hqiA* in some QS-regulated phenotypes of *P. carotovorum.* *P. carotovorum* CECT 225^{T} (1), *P. carotovorum* (pME6010) (2) and *P. carotovorum* (pME6010::*hqiA*) (3) tested in pectin, casein, DNase, PVK (alkaline phosphatase), swarming and swimming media.
